# Seasonal variations on endothelium‐dependent flow‐mediated vasodilation in adults with type 2 diabetes and nondiabetic adults with hypertension and/or dyslipidaemia who perform regular exercise

**DOI:** 10.1002/edm2.168

**Published:** 2020-07-06

**Authors:** Hiroto Honda, Makoto Igaki, Motoaki Komatsu, Shin‐ichiro Tanaka

**Affiliations:** ^1^ Department of Physical Therapy Faculty of Health Sciences Aino University Higashioda Japan; ^2^ Department of Rehabilitation Toyooka Hospital Hidaka Medical Center Toyooka Japan; ^3^ Department of Internal Medicine Toyooka Hospital Hidaka Medical Center Toyooka Japan

**Keywords:** exercise, flow‐mediated vasodilation, type 2 diabetes

## Abstract

**Introduction:**

Endothelium‐dependent flow‐mediated dilation (FMD) of the brachial artery often changes seasonally. We aimed to examine the association between the seasonal variation on FMD and regular exercise in adults with type 2 diabetes (T2D) and nondiabetic adults with hypertension and/or dyslipidaemia (non‐T2D).

**Methods:**

This retrospective study included 14 T2D and 17 non‐T2D adults, who started to perform moderate‐intensity aerobic exercise for 30‐40 min/d at a hospital gym in 2006‐2010 and maintained exercise performance at least 2 d/wk until the end of the observation period. We observed and analysed the data for 5 years (from March 2011 to February 2016). FMD, cardio‐ankle vascular index (CAVI) and metabolic outcomes were compared among seasons in the T2D and non‐T2D groups.

**Results:**

The FMD values were lower in winter than in other seasons in both groups (all *P* < .01). The annual range of FMD was larger by 31% in the T2D group than in the non‐T2D group (*P* < .05). The systolic blood pressure (BP) values were higher in winter than in other seasons in both groups (all *P* < .01), and the diastolic BP values were higher in winter than in summer in both groups (T2D: *P* < .05; non‐T2D: *P* < .01). CAVI and other outcomes did not change seasonally.

**Conclusions:**

Flow‐mediated vasodilation showed seasonal variation in T2D adults, even if they performed exercise regularly for a long period of time. Additionally, we found that the annual range of FMD might increase with the presence of T2D.

## Graphical abstract

Flow‐mediated vasodilation (FMD) seasonally varies in adults with type 2 diabetes and nondiabetic adults with hypertension and/or dyslipidaemia despite performing long‐term exercises. Annual range of FMD may increase with the presence of type 2 diabetes.

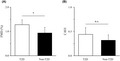

## 1 INTRODUCTION

Vascular endothelial dysfunction, which is observed in the absence of frank atherosclerosis,^1^ independently plays an important role in the onset of cardiovascular disease (CVD).^2^ Flow‐mediated dilation (FMD) of the brachial artery is often used to quantify endothelial function (EF), which indicates an endothelium‐dependent response to shear stress by using a noninvasive ultrasonographic assessment and reflects nitric oxide (NO) production.^3^, ^4^ Previous studies showed that the FMD value was considered an independent predictor of CVD.^5^, ^6^

Change in meteorological factors such as outdoor temperature and change of lifestyle are important factors influencing blood circulation.^7^ The seasonal variation on blood pressure (BP) is often observed, the value of which is higher in winter than in other seasons.^8^ Similarly, the FMD value may be affected by seasons and temperatures. A large population study (n = 2587) from the Framingham Heart Study showed that the highest and lowest FMD values were in summer and winter, respectively^7^; however, this cross‐sectional study investigated FMD in different participants among seasons. A prospective study, which examined the same individuals with hypertension, diabetes and/or dyslipidaemia, compared FMD between the warm (from July and September) and cool (from November to March) seasons.^9^ This revealed that FMD in the warm season was significantly higher than that in the cool season, similar to the above large population study.^7^

These studies investigated the seasonal variation on FMD without an analysis on the participants' exercise habits. The increases of local blood flow and cardiac output are induced by skeletal muscle contractions during exercise, and they raise the shear stress on vascular endothelium and the NO production.^10^ However, the amount of performing exercise, such as in sports and exercise therapy, often changed seasonally when people have severe climate conditions. People become less active and more sedentary in winter than in spring and summer.^11^, ^12^ Therefore, if people maintain their exercise habits throughout the year, the seasonal variations on FMD may decrease; however, research on the influence of long‐term exercise on FMD has been limited.

Moreover, we previously examined whether performing regular moderate‐intensity aerobic exercise (MIAE) (frequency, 2‐3 d/wk) for 6 months could improve FMD in adults with T2D and nondiabetic adults with hypertension and/or dyslipidaemia (non‐T2D).^13^ As a result, the FMD values improved after the intervention; however, the FMD change may be affected by the presence of T2D.^13^ In this study, we examined whether performing regular exercise could decrease the seasonal variation on FMD in T2D and non‐T2D adults.

## 2 MATERIALS AND METHODS

### 2.1 Participants

Overall, 19 T2D and 27 non‐T2D adults aged 50‐74 years started regular visits to the Toyooka Hospital Hidaka Medical Center (Toyooka, Japan) as outpatients in 2006‐2010. All patients received medical nutritional education (energy intake: 25‐30 kcal/kg/d) supervised once every 1‐2 months by dieticians, and most of them took oral hypoglycaemic, hypotensive and/or hypolipaemic agents. The medication conditions in the patients were stable with no major change (eg from oral hypoglycaemic agents to insulin injections) throughout the study period. Written informed consent was obtained from all patients. The study protocol was approved by the institutional review board of the Toyooka Hospital Hidaka Medical Center (approval number: 12) in accordance with the Declaration of Helsinki.

### 2.2 Study design and exercise protocols

This was a retrospective study that observed data from March 2011 to February 2016. The patients started to perform regular MIAE at the hospital gym at the start of their outpatient visits in 2006‐2010, and they maintained exercise performance until the end of the observation period (February 2016). The reason why we observed the data for 5 years was to avoid being affected by climate conditions that occurred in a single year. In addition, to evaluate the stable physical status, we observed the data after at least a year had passed from the start of performing exercise (March 2011).

All patients were evaluated the peak oxygen uptake (VdotO_2_peak) by a cardiopulmonary exercise test before the start of performing exercise. The test consisted of 2‐minute warm‐up and exercise using a bicycle ergometer with ramp loading of 15‐20 W/min, and the data were derived on a breath‐by‐breath basis. On either the same day of the test or within a week, the patients performed MIAE, which included walking or cycling using a treadmill or a bicycle ergometer, (50%–60% VdotO_2_peak) for ~30 minutes under the guidance of physical therapists.

After the first exercise experience, the patients performed regular MIAE using a treadmill or a bicycle ergometer for 30‐40 min/d and at least 2 d/wk at the hospital gym until the end of the observation period. All patients were free to choose the timing of exercise from 8:30 AM to 5:00 PM The same two physical therapists supervised the exercise performed, and they monitored the heart rates and the Borg rating of perceived exertion scales,^14^ which were in the range of 11‐13, while performing exercise to check if the intensities of exercise were properly maintained. During the observation period, we asked them to perform activities of daily living as usual, except for the gym‐based exercise.

### 2.3 Measurements

We divided a year into spring (March, April, May), summer (June, July, August), autumn (September, October, November) and winter (December, January, February) and extracted values measured in the following months as representative values: April (or May), July (or August), October (or November) and January (or February). The measurements in this study were as follows: FMD, cardio‐ankle vascular index (CAVI), body mass index (BMI), waist circumference, BP, fasting plasma glucose (FPG), glycated haemoglobin (HbA1c), homeostasis model assessment of insulin resistance (HOMA‐IR), triglyceride and low‐density lipoprotein (LDL) and high‐density lipoprotein (HDL) cholesterol. HbA1c was measured and analysed only in the T2D group because that in the non‐T2D group was not always measured during the study period. The outcomes were measured in the morning after an overnight fast. We instructed the patients not to drink beverages containing caffeine and alcohol before the measurements and not to smoke on the day of the examination. On the same day, we asked the patients to answer questions regarding their lifestyle, including habits of exercise except for the gym‐based exercise (≥30 min/d, ≥2 times/wk), cigarette smoking (≥1 time/d) and alcohol drinking (≥30 g/d for males and ≥20 g/d for females).

FMD in the brachial artery and CAVI were measured in the supine position by EF18G (UNEX) and VaSera VS‐1000 (Fukuda Denshi), respectively. At least 30 minutes before the measurements of FMD, the patients entered in the examination room, the temperature of which was maintained at approximately 25°C. The patients took a rest in the supine position for 15 minutes. The brachial artery on the patient's nondominant arm was scanned by an ultrasound probe at 10 MHz. After recording the baseline image, BP cuff placed distal to the ultrasound probe was then inflated to 50 mm Hg above systolic BP for 5 minutes. Upon cuff release, the postdeflation arterial image was captured until 120 seconds to determine peak dilation. The baseline and maximal postdeflation diameter of the brachial artery were measured. FMD was defined as the relative change in the maximal postdeflation diameter, compared to the preocclusion diameter (%FMD).The measurements of FMD and CAVI were taken by two trained examiners.

Additionally, we extracted the climate data in the study area (Toyooka, Japan) from the Japan Meteorological Agency.^15^ The measurements were as follows: outdoor temperature, daylight time and rainfall.

### 2.4 Exclusion criteria and extraction in patients

**FIGURE 1:**
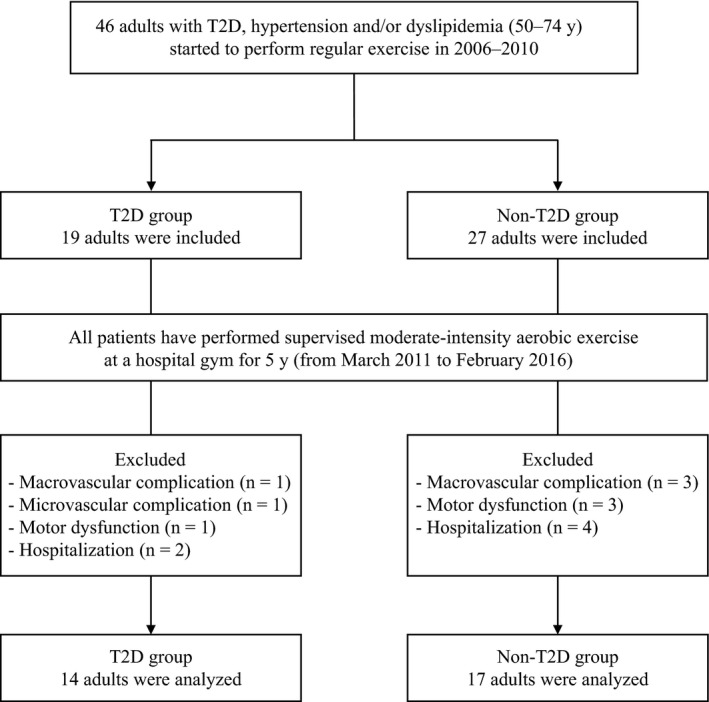
Flow chart of the study population selection, including patient recruitment. non‐T2D, nondiabetes and hypertension and/or dyslipidaemia; T2D, type 2 diabetes

The patients corresponding to the following criteria for 5 years were excluded from the analysis: macrovascular complications (coronary artery disease, peripheral arterial disease and stroke), microvascular complications (diabetic nephropathy, neuropathy and retinopathy), motor dysfunctions and other diseases affecting physical activity. Hence, 31 patients were finally included and analysed (Figure 1). Fourteen patients were in the T2D group, while 17 were in the non‐T2D group.

### 2.5 Statistical analysis

All values are reported as the mean ± standard deviation. The variables at the start of the observation (baseline) between the T2D and non‐T2D groups were compared using Fisher's exact test for nominal variables and independent t test for continuous variables. The mean values of each season throughout the 5‐year observation periods were calculated. One‐way repeated measures analysis of variance (ANOVA) was performed to analyse the outcomes among seasons, followed by Tukey's post hoc test. Furthermore, we calculated the annual ranges of the FMD and CAVI values, based on the subtraction of minimum value from maximum value in each year, and the differences in those between the two groups were compared using independent *t* test. All analyses were performed using the IBM SPSS statistics (version 20.0; IBM). The statistical significance was set at *P* < .05.

## 3 RESULTS

### 3.1 Characteristics

The baseline characteristics of the analysed patients are shown in Table 1. There were significant differences between the T2D and non‐T2D groups in the FPG, systolic BP, diastolic BP, triglyceride, LDL‐cholesterol and HDL‐cholesterol at baseline (*P* < .05, *P* < .01, *P* < .01, *P* < .05, *P* < .05 and *P* < .05, respectively). No patients had regular exercise habits except for the gym‐based exercise.

**TABLE 1:** Characteristics of the study patients at baseline

Variables	Total (n = 31)	T2D (n = 14)	Non‐T2D (n = 17)	*P*‐value
Gender (male/female)	12/19	5/9	7/10	.99
Age (y)	66.3 ± 4.4	65.9 ± 3.2	66.7 ± 4.1	.41
Duration of T2D (y)	—	12.9 ± 2.1	—	—
Hypertension (n) (%)	17 (54.8)	6 (42.9)	11 (64.7)	.29
Dyslipidaemia (n) (%)	21 (67.7)	9 (64.3)	13 (76.5)	.69
Cigarette smoking (n) (%)	5 (16.1)	2 (14.3)	3 (17.6)	.99
Alcohol drinking (n) (%)	7 (22.6)	2 (14.3)	4 (23.5)	.66
Exercise habits except for gym‐based exercise (n) (%)	0 (0.0)	0 (0.0)	0 (0.0)	—
FMD (%)	4.3 ± 1.1	4.2 ± 1.2	4.4 ± 0.9	.08
CAVI	8.7 ± 1.3	8.6 ± 1.3	8.7 ± 1.5	.12
BMI (kg/m^2^)	23.9 ± 3.4	24.0 ± 3.4	23.8 ± 4.3	.24
Waist circumference (cm)	89.5 ± 8.1	90.1 ± 7.8	88.8 ± 8.6	.24
FPG (mg/dL)	102.5 ± 11.8	103.6 ± 12.4	101.5 ± 10.2	.04
HbA1c (%) (mmol/mol)	—	7.1 ± 0.7 (53.7 ± 7.2)	—	—
HOMA‐IR	1.8 ± 0.8	1.8 ± 0.5	1.7 ± 0.9	.12
Systolic BP (mm Hg)	133.0 ± 14.7	130.3 ± 15.9	136.1 ± 12.1	<.01
Diastolic BP (mm Hg)	75.2 ± 9.8	73.3 ± 7.9	76.8 ± 9.6	<.01
Triglycerides (mg/dL)	130.9 ± 11.5	129.0 ± 10.3	132.5 ± 11.6	.03
LDL‐cholesterol (mg/dL)	133.2 ± 12.2	131.0 ± 11.6	134.9 ± 12.4	.03
HDL‐cholesterol (mg/dL)	54.2 ± 9.1	55.8 ± 9.2	52.9 ± 8.4	.01

Note

Values are presented as mean ± standard deviation.

*P*‐value: T2D group vs Non‐T2D group, Fisher's exact test or independent t test.

Abbreviations: BMI, body mass index; BP, blood pressure; CAVI, cardio‐ankle vascular index; FMD, flow‐mediated vasodilation; FPG, fasting plasma glucose; HbA1c, glycated haemoglobin; HDL, high‐density lipoprotein; HOMA‐IR, homeostasis model assessment of insulin resistance; LDL, low‐density lipoprotein; non‐T2D, nondiabetes and hypertension and/or hyperlipidaemia; T2D, type 2 diabetes.

### 3.2 Seasonal variations on outcomes

Regarding the climate data during the 5‐year observation period in this study area, the mean outdoor temperature, daylight time and rainfall in winter were 3.7°C, 63.5 h/mo and 241.7 mm/mo, respectively, which differed from those in summer (25.3°C, 162.4 h/mo and 149.3 mm/mo, respectively).

**FIGURE 2:**
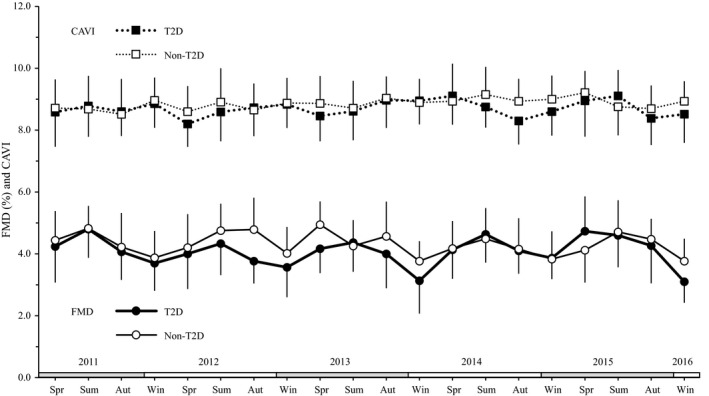
Longitudinal changes of flow‐mediated vasodilation (FMD) and cardio‐ankle vascular index (CAVI) for 5 y. Values are presented as mean ± standard deviation. Aut, autumn; non‐T2D, nondiabetes and hypertension and/or dyslipidaemia; Spr, spring; Sum, summer; T2D, type 2 diabetes; Win, winter

**FIGURE 3:**
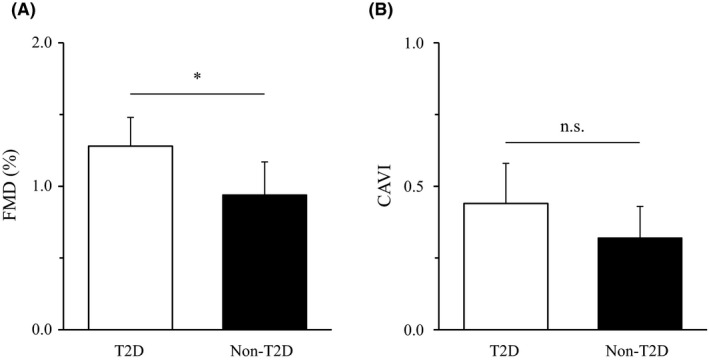
Comparisons of annual ranges of flow‐mediated vasodilation (FMD) (A) and cardio‐ankle vascular index (CAVI) (B) between the type 2 diabetes (T2D) and nondiabetes and hypertension and/or dyslipidaemia (non‐T2D) groups. Values are presented as mean ± standard deviation. n.s., statistically nonsignificant difference. *P < .05

The longitudinal changes of FMD and CAVI for 5 years are shown in Figure 2. The seasonal variations on the outcomes in the T2D and non‐T2D groups are shown in Table 2. The one‐way ANOVA showed significant differences in FMD among seasons in both groups (both *P* < .01), and the FMD values were lower in winter than in other seasons (all *P* < .01). The annual range of FMD was larger by 31% in the T2D group than in the non‐T2D group (1.3 ± 0.2% vs 0.9 ± 0.2%, *P* < .05; Figure 3A). Conversely, the differences among seasons were not significant in CAVI in both groups, and there was no significant difference in the annual range of CAVI between the groups (0.4 ± 0.1 vs 0.3 ± 0.1, *P* = .31; Figure 3B).

**TABLE 2:** Comparisons of outcomes among seasons

Variables	Group	Spring	Summer	Autumn	Winter	*P*‐value
FMD (%)	T2D	4.3 ± 1.0^‡‡^	4.5 ± 0.8^†^, ^‡^	4.1 ± 1.1^‡‡^	3.5 ± 1.1	<.01
	Non‐T2D	4.5 ± 1.1^‡‡^	4.6 ± 0.7^‡‡^	4.4 ± 0.9^‡‡^	3.9 ± 0.6	<.01
CAVI	T2D	8.8 ± 1.5	8.8 ± 1.1	8.6 ± 1.0	8.7 ± 0.9	.61
	Non‐T2D	8.8 ± 1.1	8.9 ± 1.3	8.8 ± 1.4	8.8 ± 1.0	.52
BMI (kg/m^2^)	T2D	24.0 ± 3.5	23.9 ± 3.2	24.0 ± 3.2	23.9 ± 2.9	.32
	Non‐T2D	23.8 ± 3.7	23.7 ± 4.1	23.8 ± 3.6	23.9 ± 3.1	.08
Waist circumference (cm)	T2D	90.0 ± 8.8	89.7 ± 6.9	89.9 ± 7.5	89.8 ± 7.2	0.25
	Non‐T2D	89.0 ± 7.7	89.2 ± 8.5	89.1 ± 7.9	89.3 ± 6.1	.12
FPG (mg/dL)	T2D	103.2 ± 11.5	102.9 ± 12.5	102.8 ± 13.1	103.7 ± 11.7	.31
	Non‐T2D	101.2 ± 10.1	101.5 ± 9.5	101.2 ± 11.5	101.9 ± 8.6	.18
HbA1c (%) (mmol/mol)	T2D	7.3 ± 0.9 (56.4 ± 7.5)	7.2 ± 0.7 (54.9 ± 7.2)	7.2 ± 0.7 (54.8 ± 7.2)	7.3 ± 0.6 (55.9 ± 6.4)	.22
	Non‐T2D	—	—	—	—	—
HOMA‐IR	T2D	1.7 ± 0.6	1.8 ± 0.6	1.8 ± 0.4	1.8 ± 0.5	.48
	Non‐T2D	1.7 ± 0.5	1.7 ± 0.5	1.7 ± 0.7	1.7 ± 0.6	.69
Systolic BP (mm Hg)	T2D	129.8 ± 12.5^‡‡^	127.0 ± 10.1^‡‡^	128.8 ± 16.3^‡‡^	133.7 ± 14.0	<.01
	Non‐T2D	131.8 ± 11.2**, ‡	127.5 ± 14.0^‡‡^	129.1 ± 15.1^‡‡^	134.9 ± 10.9	<.01
Diastolic BP (mm Hg)	T2D	72.6 ± 7.2	72.2 ± 8.3^‡^	72.9 ± 10.1	73.8 ± 9.7	.04
	Non‐T2D	76.2 ± 9.2	75.5 ± 10.0^‡‡^	76.8 ± 7.9	77.6 ± 8.2	<.01
Triglyceride (mg/dL)	T2D	129.7 ± 13.2	131.8 ± 9.9	130.4 ± 8.4	130.7 ± 12.4	.47
	Non‐T2D	132.0 ± 10.2	131.2 ± 12.9	132.9 ± 10.7	132.5 ± 11.1	.42
LDL‐cholesterol (mg/dL)	T2D	130.7 ± 11.6	130.3 ± 14.4	130.0 ± 14.0	131.0 ± 9.6	.93
	Non‐T2D	136.1 ± 11.1	133.6 ± 13.9	134.2 ± 12.9	135.7 ± 13.8	.20
HDL‐cholesterol (mg/dL)	T2D	56.1 ± 9.3	56.0 ± 10.2	55.8 ± 9.1	55.7 ± 11.0	.89
	Non‐T2D	53.1 ± 11.1	53.4 ± 9.9	53.1 ± 9.3	52.9 ± 10.1	.97

Note

Values are presented as mean ± standard deviation.

*P*‐value: the difference among seasons, one‐way repeated measures analysis of variance.

BMI, body mass index; BP, blood pressure; CAVI, cardio‐ankle vascular index; FMD, flow‐mediated vasodilation; FPG, fasting plasma glucose; HbA1c, glycated haemoglobin; HDL, high‐density lipoprotein; HOMA‐IR, homeostasis model assessment of insulin resistance; LDL, low‐density lipoprotein; non‐T2D, nondiabetes and hypertension and/or hyperlipidaemia; T2D, type 2 diabetes.

** *P* < .01 vs corresponding summer in the same group.

^†^ *P* < .05 vs corresponding autumn in the same group.

^‡^ *P* < .05, ^‡‡^ *P* < .01 vs corresponding winter in the same group.

With respect to systolic and diastolic BPs, the one‐way ANOVA showed significant differences among seasons in both groups, and those in winter were the highest (Table 2). Regarding other variables, there were no variations among seasons.

## 4 DISCUSSION

This is the first study that examined the association between the seasonal variation on FMD and performing regular exercise for a long period of time, which included supervised gym‐based exercise, in adults with T2D and non‐T2D adults. We found that the FMD values changed seasonally in both groups despite maintaining exercise habits. Moreover, the annual range of FMD in T2D adults was larger than that in non‐T2D adults.

Systolic and diastolic BPs are considered independent determinants of FMD.^16^ Increased BP impairs EF and causes decreased FMD, especially in winter.^7^ Moreover, the degree of dysfunction of EF may correlate with the severity of hypertension.^17^ The most likely explanations for the BP change are the variations on climate factors and lifestyle. Regarding the climate factors, the exposure to low outdoor temperature induces arteriolar vasoconstriction,^8^, ^18^ and the decrease in vitamin D synthesis in winter causes an increase of BP due to low sun exposure and inhibition of the renin‐angiotensin system.^18^ In Toyooka City, outdoor temperature and daylight time in winter during the observation period were 3.7°C and 63.5 h/mo, respectively, which were lower and shorter than those in the general Japanese regions (eg 6.5°C and 177.0 h/mo, respectively, in Tokyo during the same period).^15^ Thus, in the present study, there were seasonal variations on BP in the T2D and non‐T2D groups, and higher BP levels in winter might cause higher FMD levels.

Performing regular exercise, which increases the endothelium‐derived NO production and bioavailability,^19^ can improve the vascular EF measured by FMD. A meta‐analysis showed that aerobic exercise (AE) (20‐60 min/session, 3‐5 times/wk) and combined AE and resistance exercise (RE) (60 min/session, 3 times/wk) significantly improved FMD in T2D patients.^20^ Additionally, the study showed that there was no significant difference in increased FMD between high‐intensity interval AE (90%–95% heart rate max or 80%–85% VdotO_2_peak) vs MIAE (60%–65% VdotO_2_peak) by two randomized control trials.^20^ In the present study, both groups performed MIAE for at least 30 min/d and 2‐3 d/wk; thus, the patients might be slightly less active than the above study population, although the intensity of exercise performed may be appropriate. Conversely, a recent study showed that moderate‐to‐vigorous physical activities such as exercises even for <10 minutes were related to improved resting BP and cardiovascular risk assessed by the Framingham Cardiovascular Disease Risk Score.^21^ Therefore, we consider that the intensity and volume of exercises in this study were sufficient for the management of FMD; however, it could not suppress the seasonal variation.

In this study, the FMD values varied seasonally, whereas the changes of BMI, FPG, HbA1c (measured only in the T2D group), HOMA‐IR and lipid profiles were not observed throughout all seasons. The above meta‐analysis also showed that the change of FMD responding to exercises occurred without the changes of BMI or glycaemic control^20^; thus, the seasonal variations on FMD might be observed without the changes of the metabolic outcomes in this study. A systematic review and meta‐analysis among 13 cohorts targeted T2D patients and reported that there was a significant difference in increased FMD due to exercises between low and high baseline BMI levels (<30 and ≥30 kg/m^2^), although there was no significant difference between ages <60 and ≥60 and between the exercise periods for 8 weeks and ≥12 weeks.^22^ Most patients in this 5‐year observation study were aged ≥60 years and not obese (<30 kg/m^2^). In summary, we consider that the seasonal variations on FMD while performing regular exercise may occur regardless of age, BMI, metabolic profiles and exercise period.

The annual ranges of FMD in the T2D and non‐T2D groups were 1.3% and 0.9%, respectively. The ranges were similar to a past study that investigated the seasonal variations on FMD in the same participants with T2D, hypertension and/or dyslipidaemia (mean value, 1.0%).^9^ As mentioned above, performing exercise induces a positive effect on EF in T2D patients; however, the response of EF to regular exercise may be weakened in T2D patients compared to nondiabetic patients.^20^ Persistent hyperglycaemia, increased circulating advanced glycation end products, or reactive oxygen species that occur in T2D patients may be related to the weakness.^23^, ^24^ A stimulation of muscle contractions for EF, which intrinsically increase vascular nitric oxide bioavailability, may be neutralized by the presence of T2D.^25^ The above meta‐analysis showed that the magnitude of the improvements in FMD by performing exercise was smaller in T2D patients than in nondiabetic patients.^20^ Thus, in this study, the difference in the annual ranges of FMD between the T2D and non‐T2D groups was observed due to the weakened effect by the presence of T2D.

In this study, the CAVI value did not change seasonally in both groups. CAVI is a parameter of systemic arterial stiffness and independent of BP.^26^ A previous Japanese study showed that CAVI did not correlate with FMD.^27^ Moreover, we previously reported that performing regular MIAE for 6 months improved FMD without the change of CAVI in adults with T2D, hypertension and/or dyslipidaemia.^13^ Therefore, FMD is a useful marker of early arteriosclerosis,^27^ and the seasonal variation on FMD might be observed without the variations on CAVI.

There were some limitations to this study. First, this was a single‐centre study, which recruited uncomplicated patients and results in small samples. Second, the results of this study might be affected by the dietary habits and medications during the observation period and characteristics of the study patients. A previous Japanese study using a self‐administered diet history questionnaire showed that the consumption of high‐energy intake in winter was higher by 254 kcal/d in spring and 156 kcal/d in summer.^28^ Therefore, the influence of dietary habits is unclear, although there were no changes in the metabolic outcomes, except for BP, which were affected by the intake of high‐energy foods. In addition, the influence of medications, especially hypotensive agents, might have been involved, as BP affected the FMD value.^16^ Therefore, further studies, such as a prospective study with larger samples and recording of dietary habits, are needed to confirm the clinical relevance of performing regular exercise in T2D and non‐T2D patients.

In summary, we found that there was a seasonal variation on FMD in adults with T2D and non‐T2D adults despite performing moderate‐intensity aerobic exercise for 30‐40 min/d and at least 2 d/wk for a long period of time. Additionally, the annual range of FMD was larger in T2D adults than in non‐T2D adults. Although further studies are needed, we propose that it may be important to consider these characteristics when managing T2D, hypertension and dyslipidaemia.

## CONFLICT OF INTEREST

The authors declare no conflicts of interest.

## AUTHOR CONTRIBUTIONS

HH contributed to the study conception and design, acquisition of data, analysis and interpretation of data, and drafting and revising the manuscript. MI contributed to the study conception and design, acquisition of data, analysis and interpretation of data, and revising the manuscript. MK and ST contributed to acquisition of data, analysis and interpretation of data, and revising the manuscript. All authors approved the final version of the manuscript.

## Data Availability

All data relevant to the study are included in the article.
